# Prospective study of Hepatitis E Virus infection among pregnant women in France

**DOI:** 10.1186/1743-422X-11-68

**Published:** 2014-04-09

**Authors:** Christophe Renou, Vincent Gobert, Christophe Locher, Abdelouahab Moumen, Oumar Timbely, Janine Savary, Anne-Marie Roque-Afonso

**Affiliations:** 1 Hôpital de Jour, Centre Hospitalier d’Hyères, Hyères 83407, France; 2 Maladies de l’Appareil Digestif, Centre Hospitalier de Meaux, Meaux 77104, France; 3 Gynécologie-Obstétrique-Maternité, Centre Hospitalier de Meaux, Meaux 77104, France; 4 AP-HP, Hôpital Paul Brousse, Virologie, Villejuif 94804, France; 5 Univ Paris-Sud, UMR-S 785, Villejuif 94804, France; 6 INSERM U785, Villejuif 94804, France

**Keywords:** HEV, Seroprevalence, Pregnancy, France

## Abstract

**Background:**

Hepatitis E Virus (HEV) infection has a poor prognosis among pregnant women from high endemic countries. HEV-prevalence and incidence among pregnant women is unknown in high-income countries such as France. This prospective study was conducted to assess HEV infection in this setting.

**Findings:**

An overall HEV prevalence of 7.74% was observed among 315 pregnant women. Seroprevalence was higher in south than in north of France (29.3% vs. 3.6%, p < 0.0001), and women with detectable IgG were older. No IgG seroconversion or IgM detection were observed during pregnancy.

**Conclusions:**

Data suggest that HEV infection is a rare occurrence during pregnancy even in regions of western countries with high seroprevalence rates.

## Background

Hepatitis E virus (HEV) infection is associated with two distinct patterns of disease [1]. In low income countries with poor sanitation and hygiene, HEV is a common cause of acute hepatitis, and is responsible for waterborne outbreaks and sporadic cases due to genotype 1 or 2 that exclusively infect humans. Disease has a high attack rate in young adults and is particularly severe among pregnant women where the mortality secondary to symptomatic infection was estimated tenfold higher than in men or non-pregnant women [2]. In high income countries, HEV is responsible for sporadic cases due to genotypes 3 and 4 that also infect other animals, and zoonotic and food-borne transmission is suggested [1]. In these countries, the clinical presentation differs from disease in high endemic areas, including older age, more marked male predominance, higher frequency of underlying liver disease, and a lack of severe disease among pregnant women. Indeed, only few cases of hepatitis E during pregnancy have been reported [3,4] and none with severe hepatitis. The role of nutritional, immunological, and genetic factors has been suggested in the pathophysiology of fulminant HEV during pregnancy in developing countries but the distinct clinical pattern between low and high income countries is still not understood. It may reflect differences in disease biology between different HEV genotypes but also a reduced exposure to the virus because endemicity is low and/or undiagnosed asymptomatic or pauci-symptomatic infections in high income countries.

## Methods

This prospective study was conducted to assess HEV prevalence and incidence among pregnant women from two hospitals in north (Meaux) and south-east (Hyères) of France. The protocol was approved by the ethical committee of Le Kremlin Bicêtre (France) and written consent was obtained from pregnant women. Blood samples were taken at the beginning of pregnancy and in postpartum. HEV serology (IgG and IgM) was performed with the Wantai immunoassay (Beijing, China). A questionnaire listing HEV risk factors was given at the time of sampling.

## Results

Serological data were available for 315 women at the beginning of pregnancy, 255 in north and 60 in south-east France. The overall HEV prevalence was 7.74% (CI 4.7%-10.8%). Seroprevalence was higher in south than in north: 29.3% (CI 15.3%-43.2%) vs. 3.6% (CI 1.3%-5.9%). Women with detectable IgG by the Wantai assay were older: 32.5+/-5 vs. 29.4+/-5 years (p = 0.012). No IgM positivity was detected in early pregnancy. Postpartum serology was available for 140 women (44.4%), in an average of 6.4+/-1.7 months’ interval after initial sampling. No IgG seroconversion or IgM detection were observed in this second sampling.

Completed questionnaires were available at the beginning of the pregnancy in 188 cases (59.6%). No difference was observed between women with and without IgG regarding trips abroad (p = 0.5), urban vs. rural residence (p = 0.41), seafood consumption (p = 0.38), pet ownership (p = 0.18) or pork liver consumption (p = 0.14). None of the HEV IgG-positive women reported a history of liver disease.

## Discussion and conclusion

Though studies on HEV IgG seroprevalence among blood donors [5-7] or epidemiologically exposed subjects [8] are available from France, there is a lack of data with respect to pregnant women. Indeed, pregnant women are usually followed as outpatients by gynecologists and HEV is still considered as a rare and exotic disease this setting. Analysis of reported seroprevalence rates has also to take into account the use of assays with different sensitivities and the geographical origin of recruited subjects. Indeed, by using more sensitive assays, recent HEV IgG seroprevalence studies conducted in France have produced much higher results than earlier studies [7]. Significant epidemiological differences also exist within the same country: previous studies have reported seroprevalence rates among blood donors five-fold higher in south-west (16.6%) [5] than in north of France (3.2%), though using the same assay [6]. This finding was confirmed by a higher frequency of clinically apparent HEV infections in the south of France [9]. The present study confirms this north-south gradient in a population of pregnant women, with much higher rates in south-east France. Reasons for this striking difference are still unknown, and the present study failed to identify life style factors that may explain this gradient. Seroprevalence rates reported here may appear lower than those recently reported in other French populations. However, we found HEV IgG positivity associated with age and it is noteworthy from previous studies that the probability of being exposed to HEV increases with age [6,7]. Our finding of a 29.3% of HEV seroprevalence in south-east France with the Wantai assay is in line with the results from Mansuy et al. who reported a rate over 40% in blood donors from south-west France aged 28 to 37 years [7].

The high HEV seroprevalence, particularly in south of France, at the onset of the pregnancy in women who report no history of liver disease confirms that most infections are subclinical or unrecognized. Concerning the incidence of HEV infection among pregnant women in western countries, a rate of 0.67% of IgM anti-HEV was shown in a Spanish cohort during the first trimester of pregnancy [10] but no clinical symptoms and normal aminotransferases levels were reported, suggesting either false-positive results or silent forms of infection. In the present study, no markers of acute infection and no seroconversion were observed during pregnancy. This absence of infection even in an area with a significant circulation of the virus, like south-east of France may be due to the relatively short time span between samplings (6 months) but also to dietary measures taken by pregnant women to prevent infectious diseases such as toxoplasmosis that could have protect them also from HEV. However, the number of questionnaires returned at the end of pregnancy was too small to assess this hypothesis.

Finally, this prospective study assessed for the first time the HEV prevalence and incidence during pregnancy in France and suggests that this infection is a rare occurrence during pregnancy, even in high endemic regions of western countries.

## Competing interests

The authors declare that they have no competing interests.

## Authors’ contributions

CR participated in the study design, has included patients and drafted the manuscript. VG managed the database. CL, AOM and OT have included patients. JS carried out the immunoassays. AMRA participated in the study design, performed statistical analyses and helped to draft the manuscript. All authors read and approved the final manuscript.
